# Comparison of Psychological Status and Investment Style Between Bitcoin Investors and Share Investors

**DOI:** 10.3389/fpsyg.2020.502295

**Published:** 2020-11-17

**Authors:** Hee Jin Kim, Ji Sun Hong, Hyun Chan Hwang, Sun Mi Kim, Doug Hyun Han

**Affiliations:** Department of Psychiatry, Chung-Ang University Hospital, Seoul, South Korea

**Keywords:** bitcoin, cryptocurrency, fear of missing out, character, temperament

## Abstract

Bitcoin has unique characteristics that have inspired people to invest in it as well as distinct drawbacks. With a rapid increase in Bitcoin prices in the short term, more investors enthusiastically began investing in it, raising concerns about a speculative bubble. This study investigated the multiple factors involved in the Bitcoin craze despite concerns about its shortcomings. In what concerns to personality traits and psychological states, online use patterns, and investment patterns, we first hypothesized that Bitcoin investors would show differences in multiple factors when compared to share investors. Based on our assumptions about these differences, we secondly hypothesized that investors’ personality, psychological states, and investment patterns could predict whether they would invest in Bitcoin or shares. In total, 307 respondents completed the research protocol and were sorted into Bitcoin investors (*n* = 101), share investors (*n* = 102), and non-investors (*n* = 104). A self-report questionnaire on demographic data, online use patterns, investment patterns as well as the Fear of Missing Out (FoMO) scale, Temperament and Character Inventory-Revised-Short (TCI-RS), Mood Disorder Questionnaire (MDQ), trait anxiety part of the State-Trait Anxiety Inventory (STAI-T), and the Korean version of the Canadian Problem Gambling Index (K-CPGI) were administered. The results of this study indicated that Bitcoin investments can be attributed to the interaction of multiple factors, among which personality, psychological states, and investment patterns are particularly important. Specifically, the investment pattern is the strongest predictive factor for Bitcoin investment. Bitcoin investors were distinct with regard to higher novelty seeking, higher gambling tendencies, and unique investment patterns. Thus, personality, psychological states, and investment patterns could explain the substantial investments in Bitcoin.

## Introduction

Bitcoin and a series of similar cryptocurrencies have fascinated people since they were first launched in 2009 following the advent of blockchain technology (Nakamoto, 2008; Vigna and Casey, 2015). Bitcoin, a new peer-to-peer (P2P) digital currency, is characterized by user anonymity, low transaction costs, security and control as well as the option of mobile payments via the Internet (Böhme et al., 2015; Yelowitz and Wilson, 2015). However, its disadvantages include high liquidity and a process that is still under development (Brière et al., 2015; Polasik et al., 2015). Bitcoin was expected to enable operational confidentiality and transparency that are independent from a central organization in its early days (Glaser et al., 2014; Pagliery, 2014; Tschorsch and Scheuermann, 2016), but it gained exceptional popularity globally as a means of escaping from the original monetary system, illegal transactions, money laundering, tax evasion, and speculation (Pichet, 2017). The involvement of speculators who regard Bitcoin as a means of investment has fueled the rapid growth of the cryptocurrency market (Bohr and Bashir, 2014). In the second half of 2017, Bitcoin price skyrocketed to almost USD 20,000, from its worth of only USD 0.06 in July 2010 (Lu, 2018; Novotný, 2018). Because of the rapid expansion and volatility of the Bitcoin market, many financial experts and regulatory agencies have warned investors about the possibility of a Bitcoin market crash (Dale et al., 2005). Nevertheless, many investors around the world dove headlong into the Bitcoin market, and eventually, the market price of Bitcoin plunged to USD 4,000 in December 2018 (Lu, 2018).

This study sought to identify which individual factors might have led investors to Bitcoin investments, despite the aforementioned shortcomings and concerns about Bitcoin. We particularly tried to focus on the investors’ psychological aspects. Investor psychology is particularly important in the Bitcoin market, since investors’ behaviors are determined by the expected profits due to the lack of intrinsic value of Bitcoin (Kristoufek, 2013). To the best of our knowledge, this is the first study conducted on Bitcoin investment psychology.

Previous studies have shown that Bitcoin investors have significantly different characteristics compared to general investors, such as younger age (Bohr and Bashir, 2014), irrational optimism over easy wealth (Pezzani, 2018), higher risk propensity (Conlon and McGee, 2020), and the psychology of *fear of missing out* (FoMO) (Pichet, 2017). Pezzani (2018) highlighted that Bitcoin investors’ irrational beliefs about its perpetual growth has led them to overvalue the margin derived from speculation. Risk propensity, which is in line with sensation-seeking, is a willingness to risk more in a period of high volatility because an investor finds trading entertaining and stimulating (Mai, 2019). A risk-seeking investor would experience more excitement in Bitcoin investment owing to its fast-paced, variegated environment (Mai, 2019). FoMO refers to a pervasive concern that someone might be having rewarding experiences that they are missing out on; in other words, a desire to stay connected with what others are doing (Przybylski et al., 2013). Pichet (2017) reported that the FoMO is the main factor creating the speculative bubble as the media buzz accentuates the global demand for Bitcoin. The interest in Bitcoin measured by search queries on Google Trends and frequency of visits on the Wikipedia was correlated with Bitcoin prices (Kristoufek, 2013). The number of tweets regarding Bitcoin was an important driver in the next day’s Bitcoin market trading volume as well as in realized volatility (Shen et al., 2019). Engaging in online forums related to Bitcoin positively predicted Bitcoin accumulation (Bohr and Bashir, 2014). In addition, user sentiments from social media and online portals contributed to Bitcoin prices (Kristoufek, 2013; Kinderis et al., 2018; Rahman et al., 2018). As such, social networking service (SNS) and web portals had a great influence on Bitcoin prices and trading, so we assumed that apart from FoMO, the online use pattern might be related to excessive Bitcoin investments. Market properties such as easy online access, simple subscription and certification procedures, and 24 h openness, influence investors’ motives as well. An earlier study of online trading discovered that the adoption of online trading was predominant in young risk-taking investors (Li et al., 2002).

However, since there has been little integrated research on factors influencing Bitcoin investors, we reviewed studies on the share market, which is popular, long established, and most comparable to the Bitcoin market. It would be possible to extract the characteristics of Bitcoin investors by comparing the differences between Bitcoin investors and share investors based on the factors that affect share investors. Factors that were identified as affecting investment behavior in the share market included age, gender, education, marital status, income, social interactions, investor’s personality, desire to become rich quickly, gambling tendency, dividends paid, expected dividends, condition of financial statements, broker recommendation, past performance of the firm share, firm’s status in an industry, current economic indicators, and share marketability (Hong et al., 2004; Lee et al., 2010; Granero et al., 2012; Shafi, 2014; Zhang et al., 2014; Conlin et al., 2015; Ahmad, 2017; Tauni et al., 2017; Oehler et al., 2018). With regard to gender, the trading volume was typically higher among male investors than their female counterparts (Zhang et al., 2014). Like Bitcoin investments, increased social interaction has been reported to facilitate share market participation (Hong et al., 2004). Several studies have suggested that investors’ personalities such as openness, extraversion, agreeableness, and neuroticism of the Big Five, or the traits of Cloninger’s Temperament and Character Inventory (TCI), such as novelty seeking, harm avoidance, and reward dependence affect investment decisions (Zhang et al., 2014; Conlin et al., 2015; Tauni et al., 2017; Oehler et al., 2018). Granero et al. (2012) examined the stock market investments involved in gambling problems. Furthermore, prior studies suggest that investment strategies, perceived profitability, and macroeconomic factors affect investment decisions (Lee et al., 2010; Ahmad, 2017).

Taken together, the probable contributing factors to Bitcoin and share investments discussed above are multi-dimensional and do not appear to be completely independent of each other. For a more comprehensive understanding, we classified the variables into demographic data, personality and psychological states, online use patterns, and investment patterns and tried to evaluate the impact of variables in each dimension hierarchically.

In the comparisons of personality and psychological states, online use patterns, and investment patterns, we hypothesized that Bitcoin investors would show differences of multiple factors, compared to share investors. Based on our assumptions about these differences, we secondly hypothesized that investors’ personality, psychological states, and investment patterns could predict whether to invest in Bitcoin or shares.

## Materials and Methods

### Participant Recruitment

Study participants were recruited between April 9 and April 16, 2018. An invitation e-mail to participate in the survey was sent to all registered members of the research company (Embrain^®^, Seoul, South Korea) who were over the age of 20. Of all those contacted, 59 candidates responded to the first e-mail. Of these responders, 110 met the inclusion criteria for Bitcoin investors, 319 met the inclusion criteria for share investors, and 3,765 met the inclusion criteria for non-investors. Given that we planned to compare 32 variables in four categories between two groups (investor vs. non-investor, Bitcoin vs. share investor group), the data of 207 participants in two groups were estimated using GPower 3.1 software (effect size = 0.2, α error = 0.05, Power = 0.95) (Faul et al., 2007). Of the 5,933 respondents in the first e-mail, 110 met the inclusion criteria for Bitcoin investors. Considering about 10% missing data, we randomly selected 110 individuals each from the 319 share investors and 3,765 non-investors. Non-study personnel generated the randomization sequence and assigned a number to each of the 319 share investors and each of the 3,765 non-investors. Those with the numbers between 1 and 110 from both groups were selected. For the purpose and analysis of the current study, Embrain^®^ sent an e-mail which included self-report questionnaires to the selected 110 Bitcoin investors, 110 share investors, and 110 non-investors. Participants were asked to complete the self-report questionnaires and submit them to Embrain^®^ within 1 month. However, 9 of the 110 Bitcoin investors, 8 of the 110 share investors, and 6 of the 110 non-investors, did not complete the questionnaires. Thus, the data from the completed questionnaires of 101 Bitcoin investors, 102 share investors, and 104 non-investors were used in the analysis. Participants were compensated with USD 20 upon completion of the survey.

The inclusion criteria for Bitcoin investors were as follows: (1) age 20 and above, (2) Bitcoin investments of more than 3 months, and (3) no experience or less than 3 months’ experience in share investments. The inclusion criteria for share investors were as follows: (1) age 20 and above, (2) share investments of more than 3 months, and (3) no experience or less than 3 months’ experience in Bitcoin investment. Finally, the inclusion criteria for non-investors were as follows: (1) age 20 and above, (2) having no experience or less than 3 months’ experience in Bitcoin or share investments.

### Assessment Scales

The self-report questionnaires used in this study included questions about demographic characteristics, online use patterns, and investment patterns as well as well-known psychological measures detailed below.

#### Personality and Psychological States

##### Cloninger’s Temperament and Character Inventory (TCI)

To assess temperament and personality, Cloninger’s Temperament and Character Inventory-Revised-Short Version (TCI-RS) (Cloninger et al., 1993) was applied to the participants. TCI is a well-known tool for assessing individuals’ biogenetic characteristics (Ebstein et al., 1996; Benjamin et al., 2000; Gusnard et al., 2003; Ellison et al., 2007). Cloninger’s psychobiological model assumes that both genetic and environmental factors influence an individual’s personality, which is represented by his/her temperament and character trait. The 140-item TCI-RS is designed to measure four temperamental factors (novelty seeking, NS; harm avoidance, HA; reward dependence, RD; and persistence, PE) and three character traits (self-directedness, SD; cooperativeness, CO; and self-transcendence, ST). The Korean version of the TCI was standardized and demonstrated reliability and validity in 2007 (Min et al., 2007). Cronbach’s αs for the NS, HA, RD, PE, SD, CO, and ST are 0.83, 0.86, 0.81, 0.82, 0.87, 0.76, and 0.90, respectively (Min et al., 2007). In this study, the T-scores of the TCI-RS were used for the analysis.

##### Fear of Missing Out (FoMO) Scale

The 10-item FoMO scale (Przybylski et al., 2013) is a brief self-report tool designed to measure robust individual differences in FoMO. Items are scored on a five-point Likert scale, with “Not at all = 1” and “Extremely true of me = 5.” (e.g., “I fear others have more rewarding experiences than me;” “When I have a good time, it is important for me to share the details online”). The Cronbach’s α of the Korean version of FoMO scale was 0.77 (Joo et al., 2018).

##### Mood Disorder Questionnaire (MDQ) and State-Trait Anxiety Inventory (STAI-T)

To assess mood and anxiety, the Mood Disorder Questionnaire (MDQ) (Hirschfeld et al., 2000) and the trait anxiety part of the State-Trait Anxiety Inventory (STAI-T) (Spielberger et al., 1983) were applied, respectively. The MDQ is a widely used self-rated scale aimed at screening the risk for bipolar spectrum disorders. The MDQ’s total score ranges from 0 to 13, where a score of 7 or more suggests the presence of bipolar spectrum disorders (Hirschfeld et al., 2000). The Korean version of the MDQ has a satisfactory internal consistency (Cronbach’s α) of 0.88 (Jon et al., 2009).

The STAI comprises 40 items, 20 of which describe trait anxiety (T-anxiety; STAI-T) and the other 20, state anxiety (S-anxiety; STAI-S) (Spielberger et al., 1983). In this study, the participants were asked to respond only to the 20 STAI-T items of the Korean version. The Cronbach’s α of this version is 0.86 (Lee et al., 2008).

#### Online Use Patterns

To assess online use patterns, the number of SNS (e.g., none, one, more than two), frequency of SNS posts per day (e.g., less than one, one to ten, more than ten), and frequency of online connections per day (e.g., less than one, one to ten, more than ten) were assessed. In our study, we adopted the following definition of SNS from Wikipedia^1^ : “an online forum which people use to enhance social networks or relations with other people who share similar personal or career interests, activities, backgrounds or real-life connections. Specific examples include Facebook, YouTube, Instagram, and Twitter.”

#### Investment Patterns

To assess investment patterns, the questionnaires included questions on the participants’ experience with investment funds other than Bitcoin and shares, the total amount of investments in Bitcoin or shares, percentage of income from Bitcoin or share investments, the investment period, investment results, recent 3 months history of investment, percentage loss on an investment, percentage gain on an investment, the reason for continuous investment, the method of trade in Bitcoin or shares, intention for a long-term trade, participants’ expectations for market price, experiences of problems due to Bitcoin or share investments (economic and social problems due to investment), and gambling experiences.

##### Korean version of the Canadian Problem Gambling Index (K-CPGI)

In addition, the Korean version of the Canadian Problem Gambling Index (K-CPGI) (Kim et al., 2011) was administered to the participants. The CPGI is a useful instrument for measuring gambling problems in the general population (Ferris and Wynne, 2001). It includes indicators of social and environmental contexts of gambling and gambling problems (Ferris and Wynne, 2001). This scale was translated into Korean and indicated internal consistency and reliability (Kim et al., 2011).

### Statistical Analyses

The demographic characteristics, personality and psychological states, online use patterns, and investment patterns of the participants were analyzed using one-way analysis of variance (ANOVA) and chi-square tests. The Bonferroni *post hoc* test, with a significance level < 0.05, was used. Because of the problem of collinearity, the Durbin-Watson test and hierarchical logistic regression were used to confirm whether the variables in the questionnaires and clinical scales explained a statistically significant amount of variance in the dependent variable of Bitcoin investment.

In a multiple hierarchical regression analysis of all investors, a discrete set of hierarchical variables, with Bitcoin investor as the dependent variable, was added: demographic factors for model 1, psychological state for model 2, online use patterns for model 3, and investment patterns for model 4. The dependent variable of “Bitcoin investors” was coded as “1” and the dependent variable of “Share investors” was coded as “0.” The definitions of “Bitcoin investors” and “Share investors” coincided with the inclusion criteria above.

Hierarchical regression analysis demonstrates whether the variables of interest can explain a significant amount of variance in a dependent variable after considering all other variables. The overall fit of each logistic regression model was assessed with its χ^2^-value (model χ^2^ and step χ^2^) as well as goodness-of-fit indices (−2 log likelihood). The models χ^2^ and χ^2^ might determine the improvement observed in the model with the predictors relative to the constant-only model or the model preceding the current model. To evaluate the practical usefulness of each model, tables of classification accuracy were also used to determine the relative success of each model in predicting the correlations with Bitcoin investment. In addition to indices of the overall model fit, Nagelkerke’s *R*^2^ was evaluated as an approximate estimate of the amount of variance in the dependent variable accounted for by the model. To test whether each individual factor had a significant relationship with Bitcoin investment, Wald statistics were used. When a significant relationship was detected by the Wald test, interpretation of the coefficient was followed by determination of the odds ratio, that is, the ratio between the probability that the event (i.e., Bitcoin investment) would occur to the probability that it would not.

## Results

### Demographic Characteristics

There was no significant difference in the age, sex, education, marital status, and job status of participants in the three groups. However, the share investor group indicated a higher income compared to non-investors (Table 1).

**TABLE 1:** Comparison of demographic, personality and psychological status, and online use pattern between Bitcoin investors, share investors, and non-investors.

	Bitcoin	Share	Non-investors	Statistics
**Demographic data**				
Age	31.9 ± 4.2	32.7 ± 3.4	31.9 ± 3.5	*F* = 1.7, *p* = 0.19
Sex (male/female)	57/44	57/45	48/56	χ^2^ = 2.7, *p* = 0.25
**Education**				
High school	8	6	6	χ^2^ = 0.6, *p* = 0.97
University	76	77	79	
Graduate	17	19	19	
**Marital status**				
Single	52	42	62	χ^2^ = 8.8, *p* = 0.07
Married	49	60	42	
Job (yes/no)	86/15	89/13	89/15	χ^2^ = 0.2, *p* = 0.69
Income ($/month)	3575.2 ± 1218.4	4020.4 ± 1966.5	3292.3 ± 1812.9	*F* = 4.8, *p* = 0.01
**Personality and psychological states**				
**TCI**				
NS	39.3 ± 9.0	35.7 ± 9.8	31.9 ± 10.4	*F* = 14.8, *p* < 0.01
HA	38.6 ± 9.1	38.1 ± 11.3	35.6 ± 11.0	*F* = 4.5, *p* = 0.01
RD	42.8 ± 8.0	42.6 ± 7.5	45.6 ± 8.9	*F* = 4.4, *p* = 0.01
PE	43.5 ± 8.2	43.4 ± 8.4	41.5 ± 9.8	*F* = 1.6, *p* = 0.12
SD	44.8 ± 9.9	45.3 ± 10.6	50.5 ± 9.4	*F* = 10.1, *p* < 0.01
CO	48.4 ± 7.7	52.2 ± 8.5	56.6 ± 8.4	*F* = 19.8, *p* < 0.01
ST	26.0 ± 11.9	27.2 ± 12.0	19.4 ± 10.7	*F* = 13.6, *p* < 0.01
FoMO	22.2 ± 7.1	20.9 ± 7.4	16.9 ± 6.8	*F* = 17.9, *p* < 0.01
MDQ	5.8 ± 3.4	5.1 ± 2.9	4.7 ± 3.8	*F* = 2.8, *p* = 0.01
STAI-T	35.4 ± 6.9	34.9 ± 9.2	32.0 ± 8.2	*F* = 5.1, *p* = 0.01
**Online use patterns**				
**SNS_Use**				
No	21	24	31	χ^2^ = 5.2, *p* = 0.27
1	42	32	30	
More than 2	38	46	43	
**SNS_Post(/day)**				
Less than 1	79	90	100	χ^2^ = 15.3, *p* < 0.01
1–10	12	7	2	
More than 10	10	5	2	
**SNS_Connection(/day)**				
Less than 1	40	41	39	χ^2^ = 4.4, *p* = 0.36
1–10	41	47	39	
More than 10	20	14	26	

*Post hoc test (Bonferroni, significance level p < 0.05) between three groups, Income: Share > Non-investors, NS: Bitcoin > Share > Non-investor, HA: Bitcoin = Share < Non-investor, RD: Bitcoin = Share > Non-investor, SD: Bitcoin = Share < Non-investor, CO: Bitcoin < Share < Non-investor, ST: Bitcoin = Share > Non-investor, FOMO: Bitcoin = Share > Non-investor, MDQ: Bitcoin = Share > Non-investor, STAI-T: Bitcoin = Share < Non-investor, SNS_Post: Bitcoin > Share = Non-investor.Non-investors, No Bitcoin or share investment or less than 3 months of experience in Bitcoin or share investment; TCI, Cloninger’s Temperament and Character Inventory; NS, Novelty Seeking; HA, Harm Avoidance; RD, Reward Dependence; PE, Persistence; SD, Self-Directedness; CO, Cooperativeness; ST, Self-Transcendence; FoMO, Fear of Missing Out; MDQ, Mood disorder questionnaires; STAI-T, Trait anxiety of State-Trait Anxiety Inventory; SNS, social network service; SNS_Use, whether an individual uses a social networking service (SNS); SNS_Post, number of postings on SNS per day; SNS Connection, number of SNS log-ons per day.*

### Personality and Psychological States

Regarding the TCI analysis, there were significant differences between the investment group (Bitcoin and share) and healthy comparison subjects in most sub-group items, except for PE. In the *post hoc* test, the Bitcoin investor group displayed higher scores in NS and lower ones in CO compared to the share investor group (Table 1).

The FoMO scores of the Bitcoin and share investor groups were higher than those of the non-investors.

The investment group (Bitcoin and share) displayed higher scores in STAI-T and MDQ compared to the healthy comparison subjects (Table 1).

### Online Use Patterns

Regarding SNS use patterns, there was no significant difference in the number of SNS platforms currently in use and the frequency of online connections per day among the three groups. However, the number of SNS posts in the Bitcoin investor group was higher than that observed in the share investor group and healthy comparison subjects (Table 1).

### Investment Patterns

There was no significant difference between Bitcoin and share investor groups with regard to their experiences with other investment funds, gambling experience, trade methods, and investment plans (short- or long-term). However, K-CPGI scores, experience of loss, percentage loss, trade frequency, and problems due to investments were greater in the Bitcoin group than in the share investor group. Further, investment amounts and investment periods were lower in the Bitcoin group than in the share investor group.

The former group made continuous investments because of previous profit, whereas the latter did so for its long-term value. Moreover, the former expected that the market price of Bitcoin would decrease significantly compared to the latter (Table 2).

**TABLE 2:** Comparison of pattern of investment between Bitcoin and share groups.

	Bitcoin	Share	Statistics
Other investments (yes/no)	26/75	29/73	χ^2^ = 2.5, *p* = 0.29
Investment amount (USD)	12268.8 ± 11338.3	33426.5 ± 15028.6	*t* = −3.2, *p* < 0.01
**Investment percentage (%)**			
<30	61	57	χ^2^ = 1.7, *p* = 0.63
30–60	24	30	
60–90	8	10	
>90	8	5	
Investment periods (month)	8.9 ± 5.6	18.7 ± 7.3	t = -10.8, *p* < 0.01
**Investment results**			
Loss	52	35	χ^2^ = 6.3, *p* = 0.04
Gain	34	44	
Even	15	23	
**Percentage loss**			
<30	12	24	χ^2^ = 20.1, *p*<0.01
30–60	25	10	
60–90	15	1	
>90	0	0	
**Percentage gain**			
<30	19	33	χ^2^ = 7.7, *p* = 0.06
30–60	12	5	
60–90	1	0	
>90	3	6	
Continuous investment			χ^2^ = 19.5, *p* < 0.01
Make up for the loss	27	20	
Previous profit	38	19	
Long-term value	31	62	
Fun	4	1	
Trade methods			χ^2^ = 1.0, *p* = 0.79
Smartphone	73	74	
Desktop	27	28	
Offline	1	0	
Investment plan (short-/long-term)	39/62	35/67	χ^2^ = 0.4, *p* = 0.56
Market price expectation (increase/steadiness/decrease)	79/12/10	81/20/1	χ^2^ = 9.4, *p* < 0.01
**Trade frequency**			
Everyday	20	12	χ^2^ = 6.2, *p* = 0.04
Every week	55	48	
Every month	26	42	
Problems (yes/no)	59/42	37/65	χ^2^ = 9.9, *p* < 0.01
Gambling (/year)			
Never	50	57	χ^2^ = 1.7, *p* = 0.79
1–10	40	36	
>10	11	9	
K-CPGI	13.5 ± 1.7	11.9 ± 1.4	*t* = 7.2, *p* < 0.01

*Other investments, investment in assets other than Bitcoin and/or shares, specifically indicating investment funds, bonds, derivatives, and real estate; Investment percentage, the percentage of investment amounts of one’s total assets; Continuous investment, the reason for continuous investment; Investment plan, short- or long-term investment plan; Market price expectation, prospects for whether market prices will increase or decrease; Problems, whether an investor has problems derived from investments; Gambling, number of gambling per year; K-CPGI, the Korean version of the Canadian Problem Gambling Index.*

### Hierarchical Logistic Regression Analysis

Considering the value (2.01) of the Durbin-Watson test, there was no autocorrelation in the data set. Of the four models employed in this study, models 2 and 4 were significantly associated with Bitcoin investment.

In model 2 (model 1 + TCI, anxiety, and mood status), model χ^2^ (29.0, *p* = 0.02), and Nagelkerke’s R^2^ (0.179, 17.9% of the variance in the dependent variable of the Bitcoin investor group) indicated that the model was adequate to predict Bitcoin investment. When the practical usefulness of the model was examined based on the classification accuracy, 16 variables in model 2 enhanced the prediction accuracy of the group membership of the dependent variable to 60.4%. With the step χ^2^-value (Step χ^2^ = 19.9, *p* = 0.03), personality and psychological status were predictive factors for Bitcoin investment.

In model 4 (model 3 + investment pattern), model χ^2^ (166.8, *p* < 0.01), and Nagelkerke’s R2 (0.749, 74.9% of the variance in the dependent variable of the Bitcoin investor group) indicated that model 4 best predicted Bitcoin investment. When the practical usefulness of the model was examined based on classification accuracy, 32 variables in model 4 enhanced the prediction accuracy of group membership of the dependent variable to 86.6%. With the highest step χ^2^-value (Step χ^2^ = 137.3, *p* < 0.01), the investment pattern was the strongest predictive factor for Bitcoin investment.

According to the Wald statistics for all independent variables, higher NS, less investment percentage, short investment period, and negative investment expectation, and higher K-CPGI scores were significant predictors of Bitcoin investments compared to share investments (Table 3).

**TABLE 3:** Hierarchical logistic regression analysis model.

Independent variables	Model 1			Model 2			Model 3			Model 4		
	B	Wald	OR	B	Wald	OR	B	Wald	OR	B	Wald	OR
**Demographic factors**												
Age	−0.043	0.837	0.957	−0.030	0.273	0.971	−0.027	0.219	0.973	0.072	0.449	1.075
Sex	−0.026	0.008	0.974	−0.136	0.191	0.873	−0.124	0.157	0.883	−0.921	1.951	0.398
Education	−0.410	3.453	0.664	−0.261	1.110	0.770	−0.268	1.120	0.765	−0.408	0.767	0.665
Marital Status	−0.298	0.752	0.742	−0.214	0.348	0.807	−0.223	0.375	0.800	−0.591	0.655	0.554
Job	0.146	0.107	1.157	0.239	0.250	1.270	0.187	0.148	1.205	−0.168	0.037	0.845
Income	−0.001	1.005	0.999	−0.002	2.730	0.998	−0.002	2.494	0.998	−0.004	3.140	0.996
**Personality and psychological status**												
NS				0.062	6.582	1.064*	0.060	6.033	1.062*	0.132	7.459	1.141*
HA				0.020	0.355	1.020	0.020	0.320	1.020	−0.109	3.007	0.897
RD				0.019	0.515	1.020	0.021	0.586	1.021	−0.021	0.170	0.979
PE				−0.001	0.002	0.999	0.000	0.000	1.000	0.034	0.560	1.035
SD				0.020	0.300	1.020	0.018	0.256	1.019	−0.039	0.404	0.962
CO				−0.061	5.172	0.940*	−0.063	5.345	0.939*	−0.034	0.463	0.967
ST				−0.025	1.378	0.975	−0.024	1.246	0.976	−0.060	1.694	0.942
FoMO				0.000	0.000	1.000	−0.004	0.017	0.996	−0.045	0.628	0.956
MDQ				0.069	1.781	1.072	0.066	1.583	1.068	0.173	2.723	1.189
STAI-T				−0.019	0.374	0.981	−0.019	0.359	0.981	−0.023	0.149	0.977
**Online use pattern**												
SNS_Use							−0.138	0.323	0.871	0.130	0.089	1.139
SNS_Post							0.150	0.289	1.162	-0.552	1.373	0.576
SNS_Connection							−0.008	0.000	0.992	-0.494	0.189	0.610
**Investment pattern**												
Other investments										-0.242	0.113	0.785
Investment amounts										0.000	3.720	1.000
Investment percentages										−1.066	5.120	0.344*
Investment periods										−0.290	24.713	0.749**
Investment results										−0.669	2.275	0.512
Continuous investment										−1.004	4.388	0.366*
Trade methods										0.463	1.019	1.589
Investment plan										2.147	6.459	8.558
Market price expectation										1.720	7.692	5.583**
Trade frequency										−0.675	1.736	0.509
Problems										1.207	3.083	3.344
Gambling										0.262	0.304	1.300
K-CPGI										0.799	13.776	2.223**

**Indices**	**Model 0**		**Model 1**		**Model 2**		**Model 3**		**Model 4**			

−2LL	280.01		270.89		250.97		250.54		113.26			
Nag *R*^2^	N/A		0.059		0.179		0.181		0.749			
Step χ^2^/p	N/A		9.1/0.17		19.9/0.03		0.4/0.94		137.3/ < 0.01			
Model χ^2^/p	N/A		9.1/0.17		29.0/0.02		29.5/0.06		166.8/ < 0.01			
Class Accur	50.5		60.4		60.4		59.9		86.6			

**p < 0.01; *p < 0.05.−2LL, −2 log likelihood; Nag R^2^, Nagelkerke’s R^2^; Class Accur, Classification Accuracy; Dependent variable, Bitcoin investor group; Model 1, Demographic factors; Model 2, Model 1 + Psychological status; Model 3, Model 2 + Online use pattern; Model 4, Model 3 + Investment pattern.NS, Novelty Seeking; HA, Harm Avoidance; RD, Reward Dependence; PE, Persistence; SD, Self-Directedness; CO, Cooperativeness; ST, Self-Transcendence; FoMO, Fear of Missing Out; MDQ, Mood disorder questionnaires; STAI-T, Trait anxiety of State-Trait Anxiety Inventory; SNS, social network service; SNS_Use, whether an individual uses a social networking service (SNS); SNS_Post, number of postings on SNS per day; SNS Connection, number of SNS log-ons per day; Other investments, having investments in assets other than Bitcoin and/or shares, specifically indicating investment funds, bonds, derivatives, and real estate; Investment percentage, the percentage of investment amounts of one’s total assets; Continuous investment, the reason for continuous investment; Investment plan, short- or long-term investment plan; Market price expectation, prospects for whether market prices will increase or decrease; Problems, whether an investor has problems derived from investments; Gambling, number of gambling per year; K-CPGI, the Korean version of the Canadian Problem Gambling Index.

## Discussion

### Personality and Psychological Traits of Bitcoin Investors

ANOVA analysis revealed that investors (Bitcoin and share) had higher NS, RD, and ST scores, lower HA, SD, CO scores, higher FoMO and MDQ scores, and lower STAI-T scores than non-investors. Moreover, Bitcoin investors had higher NS scores and lower CO scores compared to share investors.

Our results are consistent with a study by Conlin et al. (2015) that showed that stock investors display distinctive personality traits such as high NS, RD, and low HA, compared to non-investors. In addition, our results are in line with previous studies suggesting that stock investors and non-investors show differences in personality traits, such as openness, extraversion, agreeableness, and neuroticism (Oehler et al., 2018; Tauni et al., 2017).

Previous research has shown that there is a correlation with the dimensions of the Big Five and the traits of the TCI (De Fruyt et al., 2000). NS, which refers to the tendency for behavioral activation and exploration when faced with novel stimuli (Cloninger et al., 1993), is positively related to the extraversion trait of the Big Five (De Fruyt et al., 2000). NS is also associated with exploratory activity in response to novel stimulation, impulsive decision-making, and sensitivity to reward cues (Cloninger et al., 1993). HA is the tendency for behavioral inhibition when faced with danger (Cloninger et al., 1993). Nervousness is an indicator of high HA, whereas being relaxed in potentially harmful situations indicates low HA (Cloninger et al., 1993). RD is the tendency to maintain previously rewarded behavior, especially with respect to signals received from others (Cloninger et al., 1993). RD is positively correlated with extraversion and openness (De Fruyt et al., 2000). Persons with high RD are motivated by emotional stimuli, are more social and interactive with others, and seek others’ approval (Cloninger et al., 1993). Conceptually, RD is likely to be associated with FoMO as well as with a higher online use. SD is an individual’s self-determination, while CO refers to the acceptance of other people (Cloninger et al., 1993). Low CO is related to social intolerance, revengefulness, opportunism (Cloninger et al., 1993), and a less agreeable personality (De Fruyt et al., 2000). ST refers to the experience of spiritual ideas (Cloninger et al., 1993). In light of these characteristics, our results show that investors are more exploratory, extraverted, impulsive, sensation, and reward seeking, less risk averse, and more influenced by sentiment and social interaction than non-investors. In addition, investors place more value on acceptance from others rather than on their own, and are more socially intolerable, revengeful, opportunistic, less agreeable, but also more spiritual.

The higher FoMO scores of investors (Bitcoin and share) compared to those of non-investors indicate that the former are more sensitive to rewarding experiences than the latter (Przybylski et al., 2013), which is consistent with previous studies suggesting that FoMO is a crucial driver in investment settings (Pichet, 2017). This is also linked to a higher RD in the TCI. The higher MDQ scores of investors over non-investors reflect investors’ higher affective instability and impulsivity (Paterniti and Bisserbe, 2018), which are conceptually linked to neuroticism of the Big Five. The lower trait anxiety scores of investors are linked to their lower HA tendency compared to non-investors (Jiang et al., 2003). FoMO, MDQ, and STAI-T scores did not differentiate Bitcoin investors from share investors.

Bitcoin investors exhibit common personality and psychological features of general share investors, but are distinguished among them by their high NS and low CO. The temperamental characteristics of Bitcoin investors may be linked to their investment styles in this study, represented by more experiences of loss, greater percentage loss, higher trade frequency, and more problems (e.g., chasing losses) due to Bitcoin investments. Oehler et al. suggested that more extraverted individuals would pay higher prices for and buy more financial assets when assets were overpriced, compared to less extraverted individuals (Oehler et al., 2018), which explains why Bitcoin investors have invested in Bitcoin despite the formation of a price bubble. Revengefulness and opportunism explain chasing losses and excessive trading. Additionally, Bitcoin investors’ higher NS and lower CO scores were the same as gamblers’ TCI profiles (Janiri et al., 2007), reflecting their gambling tendencies. The K-CPGI scores of Bitcoin investors in this study were significantly higher than those of share investors. Based on these results, we suggest that Bitcoin investors have a higher propensity to gamble than share investors. In summary, Bitcoin investors have a tendency to trade excessively, and when compared to general investors, they seem to be overly active, sensation-seeking, impulsive, opportunistic, and revengeful.

The main value of this study is the adoption of clinical scales such as TCI to compare investors and non-investors, Bitcoin investors, and share investors. Although these scales are mostly popular psychological tests in current psychiatric practice and research (46), they have never been applied to research on the Bitcoin market to the best of our knowledge.

### Investment Patterns of Bitcoin Investors

Small investment amounts, short-term investment plans, more frequent trading, more losses, and significant gambling tendencies were observed in Bitcoin investors compared to share investors in the chi-square analysis. These features of Bitcoin investors match those of day traders. Day trading refers to a highly active trading strategy based on short-term asset price movements, in which traders repeatedly buy and sell the same financial assets throughout the day to capture profits on each trade (Ryu, 2012). They use short-run contrarian strategies rather than momentum strategies (Chung et al., 2009). Although they spend more time on trading and pay more in transaction costs, they do not make higher profits than other investors do (Markiewicz and Weber, 2013). Day trading activity increases as gambling risk propensity increases (Markiewicz and Weber, 2013). Day traders in stock markets prefer lower-priced, more liquid, and more volatile stock through which they may seek greater profit opportunities (Chung et al., 2009). The Bitcoin market is expected to be more favored by day traders owing to its high volatility compared to the stock market. Indeed, crypto-day trading has been remarkable (Mai, 2019). Chasing losses—the main reason for the continued investments of Bitcoin investors who participated in this study—and problems due to investments that interfere with different areas of life, are features of excessive stock trading (Dixon et al., 2018). Such behavior is also observed during the progressive-loss phase in pathologic gambling (Sadock and Sadock, 2011; Dixon et al., 2018). The overlap with day traders and excessive traders in the stock market and gamblers support our idea that Bitcoin investors tend to engage in excessive trading behavior.

Bitcoin investors’ market price expectations were more negative than those of share investors, which we did not expect. This could be explained in several ways. This study was conducted for a month from April 2018, when Bitcoin prices had dropped by a third within 4 months. All the Bitcoin traders who participated in this study would have watched the market crash, and approximately half of them suffered huge losses. Bitcoin traders must have known that the overall market prices would fall; however, they continue their investments, expecting profits, possibly with grandiose and omnipotent fantasies that they would control whole events, which is in line with irrational optimism. Otherwise, investments continued to make up for the losses.

### Effects of Psychology and Investment Patterns on the Choice of Bitcoin Investments

The results of the hierarchical logistic regression analysis suggest that personality traits, psychological states, and investment styles are predictors of Bitcoin investments. More specifically, high NS scores, high K-CPGI scores, small investment percentages, short period of investments, chasing losses, and negative investment expectations predicted Bitcoin investments rather than share investments.

Most importantly, an individual’s investment style turned out to be an important predictor of Bitcoin investments. This could be explained by the fact that the Bitcoin market is new and emergent and, thus, highly correlated with idiosyncratic skewness, whereas the share market is long established (Novotný, 2018), giving investors a limited potential to be differentiated according to their investment strategies. The fact that the Bitcoin economic market size is still small compared to that of the share market (Al-Yahyaee et al., 2018), despite its expansion (Hileman and Rauchs, 2017), also supports this idea.

This study explored whether human characteristics such as personality, psychologic states and investment patterns, could predict Bitcoin investments. We believe the results obtained offer several contributions to the literature. First, to our knowledge, this is the first study to describe the Bitcoin market as behavioral finance based on statistical evidence. Whether the Bitcoin market is better explained by the efficient market hypothesis or behavioral finance remains to be debated (Bartos, 2015). Behavioral finance explains many observed market movements that are not explained by the efficient market hypothesis, which posits that the prices on the market reflect all known information, and change quickly in response to new information available to all investors (Bartos, 2015). According to the theory of behavioral finance, investors are affected by multiple factors and their decision-making could be irrational (Shiller, 2003). Second, it is of preventive value to identify personality traits of those who are likely to invest in Bitcoin. Knowledge about individuals’ personality and psychological traits would help them make optimal investment decisions.

### Limitations

This study had several limitations. First, the participants of this study were Koreans who played extraordinary roles in Bitcoin markets. While Korea, as the third largest Bitcoin market in terms of trade volume, accounts for almost 20% of all Bitcoin trades worldwide, the country’s economic size is much smaller than that (Yim et al., 2018). Consequently, the implications of our study could not be generalized to global investors. Second, as most participants were in their 20s and 30s, the results of this study could hardly be generalized to Bitcoin and share investors of all ages. Although we could not confirm whether age was an important confounding factor in this study, other studies have established that young people are more eager to invest in cryptocurrencies than older people (Bohr and Bashir, 2014). Third, the possibility of a reporting bias must be considered, as the collected data were fundamentally based on self-report questionnaires. Moreover, there could be non-response bias even though we tried our best to avoid it through a sufficient data collection period (1 month), by sending reminders, and ensuring confidentiality. Finally, the causal relationships among independent and dependent variables were uncertain because they were examined cross-sectionally. Further studies are needed to reveal the causality of these associations.

## Conclusion

The current results suggest that certain personality traits and psychological status, as well as investment patterns, could be associated with Bitcoin investment. In particular, the investment pattern could strongly predict Bitcoin investment. High novelty seeking, small investment percentage, short investment period, greater tendency to chase losses, negative market price expectations, and a gambling tendency, could predict Bitcoin investments vs. share investments.

## Data Availability Statement

All datasets generated for this study are included in the article/supplementary material.

## Ethics Statement

The studies involving human participants were reviewed and approved by the Chung Ang University IRB. The patients/participants provided their written informed consent to participate in this study.

## Author Contributions

DH and HK designed the study and wrote the manuscript. JH and HH conducted the statistical analysis. DH and SK wrote the manuscript. All authors contributed to and have approved the final manuscript.

## Conflict of Interest

The authors declare that the research was conducted in the absence of any commercial or financial relationships that could be construed as a potential conflict of interest.

## Footnotes

1 https://en.wikipedia.org/wiki/Social_networking_service
